# Multidrug-resistant enterobacteriaceae in coastal water: an emerging threat

**DOI:** 10.1186/s13756-020-00826-2

**Published:** 2020-10-30

**Authors:** Regev Cohen, Svetlana Paikin, Assaf Rokney, Maxim Rubin-Blum, Peleg Astrahan

**Affiliations:** 1 Head of Infectious Diseases and Infection Control Units, Sanz Medical Center, Laniado Hospital, 16 Divrei Haim St, Kiryat Sanz, 42150 Netnaya, Israel; 2 Technion University, Haifa, Israel; 3 Central Laboratories, Ministry of Health, Jerusalem, Israel; 4 Israel Oceanographic and Limnological Research, Haifa, Israel; 5 Israel Oceanographic and Limnological Research, Kinneret Limnological Laboratory, Migdal, Israel

**Keywords:** Carbapenemase producing enterobacteriaceae (CPE), Seawater, Estuary, Israel, Public health, *Enterobacter* spp.

## Abstract

**Background:**

The environmental role of carbapenemase-producing Enterobacteriaceae (CPE) acquisition and infection in human disease has been described but not thoroughly investigated. We aimed to assess the occurrence of CPE in nearshore aquatic bodies.

**Methods:**

Enterobacteriaceae were cultured from coastal and estuary water near Netanya, Israel in June and July of 2018. Bacteria were identified by VITEK2® and their antimicrobial susceptibility was tested according to the CLSI guidelines. Enterobacteriaceae genomes were sequenced to elucidate their resistome and carbapenemase types.

**Results:**

Among other clinically relevant bacteria, four CPE (three *Enterobacter* spp and one *Escherichia coli* isolate) were isolated from two river estuaries (Poleg and Alexander Rivers) and coastal water at a popular recreational beach (Beit Yanai). Molecular analysis and genome sequencing revealed the persistent presence of rare beta-lactamase resistance genes, including *bla*_IMI-2_ and a previously unknown *bla*_IMI-20_ allele, which were not found among the local epidemiological strains. Genome comparisons revealed the high identity of riverine and marine CPE that were cultivated one month apart.

**Conclusions:**

We show that CPE contamination was widespread in nearshore marine and riverine habitats. The high genome-level similarity of riverine and marine CPEs, isolated one month apart, hints at the common source of infection. We discuss the clinical implications of these findings and stress the urgent need to assess the role of the aquatic environment in CPE epidemiology.

## Background

Carbapenem-resistant Enterobacteriaceae (CRE), in particular, carbapenemase- and extended-spectrum β-lactamase-producing Enterobacteriaceae (CPE and ESBL-PE) endanger global health, as they have spread worldwide during the last two decades. The risk factors for the acquisition of these multidrug-resistant organisms (MDROs) are usually associated with healthcare [1] and overseas travel [2]. Potential modes of environmental transmission of these bacteria to humans outside of healthcare facilities following exposure to wildlife, livestock and pet animals have been reported [3]. The environmental routes of CRE infection may play a role in causing human disease and spreading it globally, however, they have not been thoroughly investigated yet. In particular, little is known about the epidemiology of CRE in the aquatic environment.

Recent studies show that CRE may contaminate aquatic environments such as marine surface water [4–7], rivers [5, 8–17], estuaries [18] and polluted drinking water [19]. Riverine CRE often harbor several carbapenemase types [8, 10, 11, 15]. A case of *bla*_IMI-2_ CP-*Enterobacter asburiae* bacteremia following a river near-drowning accident was described, and the infecting pathogen was isolated from the river one month later [16]. In two studies that reported the isolation of CPE from seawater, *Enterobacter* spp. was prominent and comprised of 69–76% of the Enterobacteriaceae isolated [6, 7]. Assessing the link between the clinical and aquatic epidemiology of CRE is often challenging. While some studies showed that clinical strains may be found in aquatic bodies [7, 9, 16], none such link was demonstrated in others [17, 20].

CRE may reach aquatic bodies as a result of organic contamination from multiple sources [21], including hospital effluents [22–24], wastewater treatment plants (WWTPs) [24–26], discharge of livestock farms and agriculture [3, 27], seepage water [28] as well as others [21]. Once mixed with the aquatic body, these effluents may introduce not only foreign MDROs, but also high doses of antibiotics, which likely trigger resistance propagation [29]. In Israel, the main sources of aquatic contamination comprise of regional councils that are not connected to WWTPs, controlled discharges from fish farms and WWTPs [30] and untreated sewage from the Palestinian Authority [31]. Israeli governmental programs that monitor the quality of coastal seawater and rivers report coliform concentrations, but not the presence of MDROs. MDRO infection, however, poses a real threat to those engaged in recreational water activities.

A young patient who suffered a near-drowning experience in one of Netanya's beaches was admitted to the Laniado medical center, Netanya, Israel, in June 2014. Bacterial screening upon his admission to the intensive care unit found he was a carrier of two CPE species: *Enterobacter cloacae* and *Klebsiella oxytoca*, both carrying *bla*_KPC_. We assumed that seawater ingestion and aspiration led to this infection, as no other risk factors could explain the high rates of CPE colonization. We hypothesized that these CPEs originated from rivers supplying water to the shoreline in the vicinity of Netanya. To test this hypothesis, we aimed to identify CPE in coastal waters and two river estuaries in the Sharon district.

## Material and methods

### Sampling sites

**Fig. 1:**
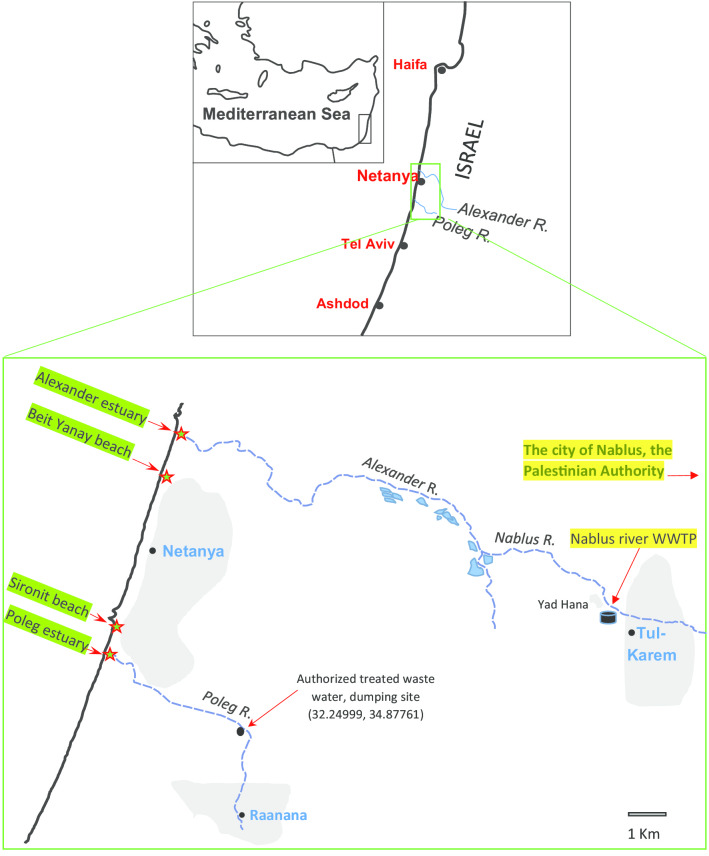
Map of the coastline near Netanya and areas of water samplings (red asterisks)

Two river estuaries were sampled in the vicinity of the Laniado hospital: Poleg (32°16′11.7"N 34°49′55.7"E) ~ 13 Km south to Laniado hospital and the Alexander River (32°23′46.7"N 34°51′57.0"E) ~ 5 Km north to the hospital. Two popular public recreational beaches along the coastline were also sampled: Beit Yanai (32°23′15.7"N 34°51′48.3"E) ~ 4.6 Km north of the hospital and 1 Km south of the Alexander river estuary; and Sironit (32°19′51.4"N 34°50′53.8"E) 1.7 Km south of the hospital and 6.5 Km north of the Poleg River estuary (Fig. 1).

### Sampling and bacterial isolation

On two occasions (June, July 2018), water samples were collected with sterile-glass containers (pre-combusted at 500 °C) and delivered to the laboratory at room temperature. 200–1300 ml of freshwater or seawater was filtered, using a vacuum pump, through a 0.2 µm sterile cellulose acetate membrane (Sartorius Stedim®). Distinct volumes of water were filtered at each site (Table 1), due to differences in turbidity and membrane clogging. Control membranes were obtained by filtering room air for the same duration. The membranes were soaked in an enrichment medium (thioglycolate broth (Novamed®)), that served as the source of cultures: first after overnight incubation at room temperature, and after 24 h of incubation at 37 °C. The media were cultured on TSBA + Blood/Chocolate agar, MacConkey agar, CNA agar, Chromagar Orientation, Chromagar MSupercarba and Streptococci Select medium plates (all from HyLabs®). Colonies were picked, isolated and then identified using VITEK2® (bioMérieux). Antimicrobial susceptibility testing was performed by VITEK2® (bioMérieux) and Etest (bioMérieux) on Mueller–Hinton agar according to the Clinical & Laboratory Standards Institute (CLSI) guidelines. Enterobacteriaceae growing on CRE plates were subjected to the modified Hodge test or CARBA NP hydrolysis. Following species isolation and identification, the carbapenemase type was identified using Cepheid® GeneXpert Carba-R for *bla*_KPC_*, bla*_NDM_*, bla*_VIM_*, bla*_IMP_*, bla*_OXA-48_ genes. The *bla*_IMI_ was identified using the polymerase chain reaction (PCR) [32].

**Table 1:** Cultivated bacteria from 4 sites during June and July 2018

Sites	Date	Water volume filtered	Bacteria cultivated			
			Carbapenem resistance	ESBL positive	Gram positive Cocci	Other bacteria
Alexander river estuary	June 2018	Stream water/750 ml	*Enterobacter asburiae bla*_*IMI*_ (genome nEC133)	NG	NG	*Escherichia coli* *Serratia marcescence* *Citrobacter sedlakii* *Pseudomonas putida*
	July 2018	Stream water/700 ml	*Enterobacter bugandensis bla*_*IMI*_ (genome nEC135)	NG	NG	NG
Poleg river estuary	June 2018	Stream water/200 ml	*Escherichia coli bla*_*OXA-48*_	*Klebsiella pneumoniae*	NG	*Enterobacter cloacae* *Acinetobacter baumannii*
	July 2018	Stream water/200 ml	NG	*Escherichia coli*	NG	NG
Beit Yanai beach	June 2018	Seawater/1300 ml	*Enterobacter bugandensis bla*_*IMI*_ (genome nEC134)	NG	*Staphylococcus aureus* *Enterococcus casseliflavus/gallinarum*	*Pseudomonas putida* *Pseudomonas luteola*
	July 2018	Seawater/1000	NG	NG	NG	NG
Sironit beach	June 2018	Seawater/950 ml	NG	NG	*Staphylococcus aureus*	*Escherichia coli* *Enterobacter aerogenes*
	July 2018	Seawater/1000	NG	NG	NG	NG
Negative control	June 2018	Room air	NG	NG	NG	NG
	July 2018	Medium/80 ml	NG	NG	NG	NG

*ESBL* Extended spectrum beta lactamase, *NG* no growth

We repeated the sampling from the same sites 3 weeks later. During the second sampling session, we mainly attempted to identify CREs, and used 60 ml of sterile media as a negative control.

### Genome sequencing, assembly and bioinformatics

DNA was extracted from three *Enterobacter* isolates: nEC133, nEC134 and nEC135 using the Presto Mini gDNA Bacteria kit (GeneAid). DNA libraries were prepared using the Illumina Nextera XT kit (Illumina). Sequencing was performed using the Illumina MiSeq platform with 2 × 250 bp paired-end reads aiming at > 100 × coverage, MiSeq Reagent Kit v2.

### Bioinformatic analyses

Sequencing was performed using the Illumina MiSeq platform with 2 × 250 bp paired-end reads aiming at > 100 × coverage, MiSeq Reagent Kit v2. Genomes were de-novo assembled with SPAdes V3.12 [33] and their quality was estimated with checkM [34]. The whole-genome multilocus sequence typing (wgMLST [35]) for *Enterobacter cloacae* complex was performed using the BioNumerics version 7.6.3 cloud-based calculation engine. wgMLST alleles were determined using assembly-free and assembly-based approaches. The resistome was identified using a standalone Resistance Gene Identifier (RGI) application with the Comprehensive Antibiotic Resistance Database (CARD [36]). Plasmid sequences were predicted with plasFlow [37] because we were unable to assemble complete plasmid sequences with PlasmidSPAdes [38]. Average nucleotide identity (ANI) was calculated as orthoANIu [39] using EzBioCloud [40]. The maximum likelihood tree of BlaIMI amino acid sequences was constructed with MEGA7 [41], based on the LG model [42].

Genomic sequences are available under the NCBI BioProject accession number PRJNA578038. The *bla*_IMI-20_ sequence was submitted to GeneBank with the accession number MN619794.

## Results

### Water-borne bacterial isolates

Enterobacteriaceae, non-fermenter bacilli and Gram-positive cocci were isolated from the coastal aquatic environment (Table 1). Four strains of CPE were found in the two sampling sessions: three isolates of CP-*Enterobacter* spp and CP*-Escherichia coli bla*_OXA-48_. The three *Enterobacter* isolates, which were isolated from seawater and freshwater, had remarkably similar antibiogram phenotype, different from that of *E. coli bla*_OXA-48_ (Table 2).

**Table 2:** Antibiograms of 4 CRE isolates cultivated from the environment

	Alexander river 1st sampling	Alexander river 2nd sampling	Beit-Yanai beach 1st sampling	Poleg river 1st sampling
Species	*E. asburiae* *bla*_IMI_ (genome nEC133)	*E. bugandensis* *bla*_IMI_ (genome nEC135)	*E. bugandensis* *bla*_IMI_ (genome nEC134)	*E. coli* *bla*_OXA48_
Drug name/MIC	MIC			
Amoxicillin/clavulanate	≥ 32	≥ 32	≥ 32	≥ 32
Piperacillin/tazobactam	≤ 4	8	8	≥ 128
Cefalexin	≥ 64	≥ 64	≥ 64	16
Cefuroxime	16	16	16	16
Cefoxitin	≥ 64	≥ 64	≥ 64	16
Ceftazidime	≤ 1	≤ 1	≤ 1	≤ 1
Ceftriaxone	≤ 1	≤ 1	≤ 1	≤ 1
Meropenem	> 32	> 32	> 32	6
Imipenem	> 32	> 32	> 32	> 32
Ertapenem	> 32	> 32	> 32	> 32
Amikacin	≤ 2	≤ 2	≤ 2	≤ 2
Gentamicin	≤ 1	≤ 1	≤ 1	≥ 16
Ciprofloxacin	≤ 0.25	≤ 0.25	≤ 0.25	≤ 0.25
Fosfomycin	64	32	64	≤ 16
Nitrofurantoin	32	32	64	≤ 16
Trimethoprim/sulfamethoxazole	≤ 20	≤ 20	≤ 20	≥ 320

*CRE* carbapenem resistant Enterobacteriaceae, *CP* carbapenemase producing, *MIC* minimal inhibitory concentration

### Resistome of aquatic *Enterobacter* revealed by genome analysis

**Fig. 2:**
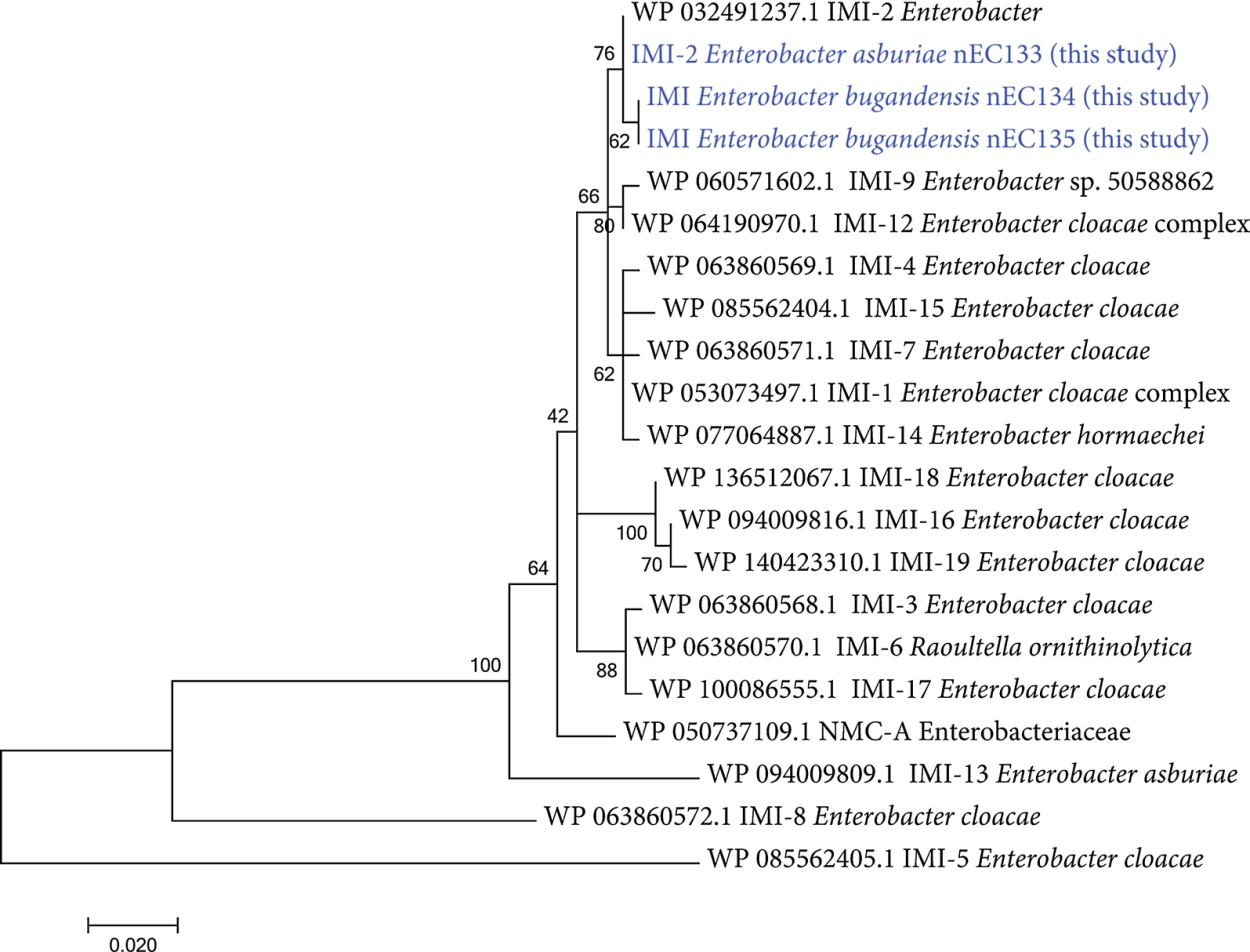
Phylogenetic tree of IMI type carbapenem-hydrolyzing class A beta-lactamases (alignment of 21 sequences of 292 amino acids). The tree is drawn to scale, with branch lengths representing the number of substitutions per site. The percentage of trees in which the associated taxa clustered together was determined based on 100 bootstrap resamples. Sequences from this study are marked in blue

We sequenced and assembled three high-quality genomes (completeness > 99%, contamination < 1%) of the aquatic *Enterobacter* isolates. nEC134 and nEC135 genomes were highly similar to each other, based both on the 99.9% ANI and 5 out of 15,612 allele differences on wgMLST analysis. These genomes were classified as *E. bugandensis* (98.7% ANI against the GenBank genomes). nEC133 genome was more diverged when compared to nEC134 and nEC135 genomes (91.5–91.6% ANI, 2600 distinct alleles), and was classified as *E. asburiae* (97.5% ANI against the GenBank genomes). The genome-derived resistomes of all the three isolates were similar and included IMI, ACT and ampC-type beta-lactamases, as well as numerous genes that encode components of antibiotic efflux pumps, and other potential antibiotic resistance mechanisms (Table 3). *E. asburiae* nEC133 *bla*_IMI_ was classified as IMI-2, based on 100% sequence identity (Fig. 2, Additional file 1: Fig. 1). *E. bugandensis* nEC134 and nEC135 carried a previously undescribed IMI-20 allele, whose sequence was distinguished from that of IMI-2 by a single nucleotide polymorphism (SNP), resulting in cysteine to phenylalanine substitution (Fig. 2, Additional file 1: Fig. 1). As in other Enterobacteriaceae, *imiR* gene that encodes a LysR-type regulator was found upstream of the *bla*_IMI_ gene [8, 43]. We were not able to assemble complete plasmids using short reads, and only 3943–6219 bp long scaffolds included the *bla*_IMI_ and *imiR* genes. However, plasFlow analysis suggested these scaffolds may be plasmid-related. The total length of scaffolds that were assigned to proteobacterial plasmids in *E. asburiae* nEC133 genome was 74,645 bp, which is comparable to that of the plasmid p3442-IMI-2 (78,374 bp). In *E. bugandensis* nEC134 and nEC135 genomes, the cumulative length of plasmid sequences was 142,368–176,174 bp, hinting at the presence of larger, or more than one plasmid. Genome assembly graphs of these isolates revealed a close linkage between the *bla*_IMI-20_ and plasmid scaffolds that carry the *traXIDTHBFNCUWICVBKEL* genes of the IncF conjugal transfer system, providing further evidence that *bla*_IMI-20_ is encoded on an IncF plasmid (Additional file 2: Fig. 2).

**Table 3:** Resistome of carbapenem-resistant Enterobacteriaceae based on genome analysis of three isolates from Alexander River estuary and Beit Yanai beach. Only results passing the strict threshold are shown

ARO term of top hit in CARD	%ID nEC133	%ID nEC134	%ID nEC135	Resistance Gene Family
IMI-2	100	97	97	IMI beta-lactamase
NmcR	96	95	95	NmcA beta-lactamase
ACT-28	91	91	91	ACT beta-lactamase
*E. coli* AmpH beta-lactamase	85	85	85	ampC-type beta-lactamase
*H. influenzae* PBP3	52	53	53	Penicillin-binding protein mutations conferring resistance to beta-lactam antibiotics
FosA4	70	70	70	fosfomycin thiol transferase
BacA**	95	95	95	undecaprenyl pyrophosphate related proteins
*E. coli* EF-Tu mutants	98	98	98	elfamycin resistant EF-Tu
AdeF	61	61	61	RND antibiotic efflux pump
AdeF*	41	41	41	RND antibiotic efflux pump
AdeF*	41	41	41	RND antibiotic efflux pump
BaeR	95	95	95	RND antibiotic efflux pump
CRP	99	99	99	RND antibiotic efflux pump
*E. cloacae* AcrA	99	99	99	RND antibiotic efflux pump
*E. coli* MarR mutant	88	89	89	RND antibiotic efflux pump
OqxA	92	92	91	RND antibiotic efflux pump
MarA	94	94	94	RND antibiotic efflux pump; General Bacterial Porin with reduced permeability to beta-lactams
RamA	95	96	96	RND antibiotic efflux pump; General Bacterial Porin with reduced permeability to beta-lactams
H-NS	96	96	96	RND/MFS antibiotic efflux pump
EmrR	93	93	93	MFS antibiotic efflux pump
*K. pneumoniae* KpnE	82	82	82	MFS antibiotic efflux pump
*K. pneumoniae* KpnF	86	86	86	MFS antibiotic efflux pump
*K. pneumoniae* KpnH	92	93	93	MFS antibiotic efflux pump
MsbA	95	95	95	ATP-binding cassette (ABC) antibiotic efflux pump

*CARD* The Comprehensive Antibiotic Resistance Database, *ARO* Antibiotic Resistance Ontology, *%ID* Percent identity of match to top hit in CARD

^*^Strict only in nEC133

^**^Distinct sequences

## Discussion

Our results suggest that CPE, as well as other MDR pathogens, occur in the coastal waters of a popular public recreational beach offshore Netanya, Israel and nearby river estuaries. These CPE appear to be abundant and well-acclimated to freshwater and seawater, as they were easy to isolate and at least on one occasion, the same *E. bugandensis* was found twice, in distant sampling sites, one month apart. It is important to state that at the time of our sampling (June and July 2018) no considerable fluctuations in seawater quality were reported [44]. Residents and tourists visiting popular recreation and leisure sites may be exposed to these MDROs.

The fact that isolates with highly similar genomes were isolated from both the riverine and marine environments, implies a mutual source, however, its origin is still unclear. Rivers are putative reservoirs and sources of MDROs, which were shown to be virulent [16, 45] and MDROs are less frequently encountered in patients drowning in seawater [46]. The pollution sources of Poleg River include discharge of urban sewage [47, 48], authorized dumping of treated sewage water [49] and cattle herding as seen on site. One of the main sources of Alexander River is the Nablus River, running from Nablus city through Tul-Karem located in the West Bank (Fig. 1). Other possible contamination sources include effluents from the adjacent WWTPs, a sea-turtles rescue center, an algae plant, an agricultural catchment basin and reservoir waters [30, 50]. Becker et al. (2013) reported that since 1995 several polluting sources were successfully treated, yet Wadi Zeimar, a major contamination source concentrating pollutants from Tul-Karem to Nablus River, remained [31]. The polluted rivers are the likely sources of marine contamination. Alternatively, coastal water can migrate up to e few kilometers inland, as shown by the daily salinity profiles measured by Ruppin Estuarine and Coastal Observatory [50], potentially reaching the possible contamination sources. Despite the limited mixing of water masses, bacterial cross-contamination may be substantial, hence bidirectional contamination is feasible.

Gut microbes may be adapted to the aquatic environment, in which their survival rates are poorly understood. Yet, cultivation-based studies demonstrate that common gut bacteria, such as Enterobacteriaceae, are frequently detected in freshwater [8, 10, 11, 15, 51, 52] and seawater [5–7]. These bacteria may be able to cope with different salinities because the human gut environment is characterized by spatial and temporal heterogeneity in osmolarity, based on the kinds of meals consumed [53]. The fitness of microbes and their growth rates depends not only on osmolarity but also on taxon-specific physiology and additional external factors such as nutrient abundance, pH and oxygen levels [54]. The three sequenced CP *Eneterobacter* spp. genomes encoded proteins that are involved in halotolerance, including the osmosensitive K^+^ channel histidine kinase KdpD, NhaA type Na^+^/H^+^ antiporter DNA-binding protein H-NS [55]. These sequences were found in scaffolds that were > 400 kbp in length and were defined as chromosomal by plasFlow. This indicates that at least some of the salt tolerance-related traits in these strains are not linked to plasmids as in other bacteria, and therefore not to *bla*_IMI_ genes, which most likely are encoded on plasmids. Salinity has been recently shown to be the most important factor modulating the distribution patterns of antibiotic resistance genes in oceans and river beach soils [56], although other studies of aquatic ecosystems failed to show this [57]. Thus, it is feasible that less-studied traits that mediate salinity tolerance may be linked to antibiotic resistance genes.

Most importantly, these aquatic isolates are only remotely associated with the local clinical epidemiology. CP *Enterobacter* spp., are uncommon in our hospital’s clinical settings: between August 2013 and February 2019, we identified 129 *Enterobacter* spp. out of 798 CRE rectal screening isolates (16%). Carbapenemase-producing *Enterobacter* spp. were detected in 65 isolates. *bla*_IMI_ genes were found only in three of these CPE isolates, while *bla*_KPC_ (50 isolates) and *bla*_NDM_ (10 isolates) were more common. However, the IMI mechanism can often go undetected, because only the five major enzymes (KPC, NDM, VIM, OXA-48 and IMP) are routinely tested. The clinical implication is that *Enterobacter* spp. carrying an unidentified *bla*_IMI_ could have been misidentified as non-CP CREs. Since patients carrying non-CP CRE isolates are not cohorted in Israeli hospitals as CPE carriers, such misidentifications increase the potential for hospital cross-infection and outbreaks.

OXA-48 has also not been frequently encountered during this period in our facility (19 isolates out of 798 CREs, mostly seen in *E. coli* spp). Nevertheless, IMI carbapenemases appear to be emerging in clinical practice [58] as well as causing nosocomial outbreaks [59].

## Conclusions

The widespread occurrence of CPE contamination in popular recreational beaches of the Sharon district is alarming and has major ramifications for environmental and public health, as well as to the public perceptions and awareness. However, occupation- or recreation-related seawater or river exposure is currently not considered a risk factor for CPE contamination, as it should probably be. It is important to consider MDROs (including CPE) when providing empiric antibiotic therapy for aspiration pneumonia during near-drowning as well as other injuries occurring during water-related activities, such as surfing and fishing.

Future large scale studies that will investigate the roles of the contamination sources along the river route to the sea, as well as resistome-related monitoring programs based on the frequent sampling of water and sediments, are crucial to mitigate aquatic CPE infection risks.

## Supplementary information

**Additional file 1**. **Figure 1**: Alignment of IMI type carbapenem-hydrolyzing class A beta-lactamase amino acid sequences (see Figure 2 in the main text for accession numbers). Names of novel IMI alleles are marked in blue, cysteine to phenylalanine substitution is shown in the frame.

**Additional file 2**. **Figure 2**: The link between *bla*_MI-20_ and *traXIDTHBFNCUWICVBKEL* genes of the IncF plasmid, based on the simplified de Bruijn assembly graph of the *E. bugandensis* nEC134 genome, generated by the SPAdes assembler and viewed in Bandage (the whole graph in the top panel and zoom in into the paths that contain the target genes in the bottom panel). The target genes are shown (*bla*_IMI-20_-red, *lysR*-yellow, *traXIDTHBFNCUWICVBKEL*-shades of blue).

## Data Availability

Genomic sequences are available under the NCBI BioProject accession number PRJNA578038. The *bla*_IMI-20_ sequence was submitted to GeneBank with the accession number MN619794.

## Abbreviations

CRE: Carbapenem-resistant enterobacteriaceae
CPE: Carbapenemase-producing enterobacteriaceae
ESBL-PE: Extended-spectrum β-lactamase-producing enterobacteriaceae
MDRO: Multidrug-resistant organism
WWTP: Wastewater treatment plants
CLSI: Clinical and Laboratory Standards Institute
PCR: Polymerase chain reaction
wgMLST: Whole-genome multilocus sequence typing
RGI: Resistance gene identifier
CARD: Comprehensive antibiotic resistance database
ANI: Average nucleotide identity
SNP: Single nucleotide polymorphism
