# Supplementary figures and images for: Multidrug-resistant enterobacteriaceae in coastal water: an emerging threat
Source: Antimicrob Resist Infect Control. 2020 Oct 30;9:169. doi: 10.1186/s13756-020-00826-2 (PMC7602311; doi:10.1186/s13756-020-00826-2)

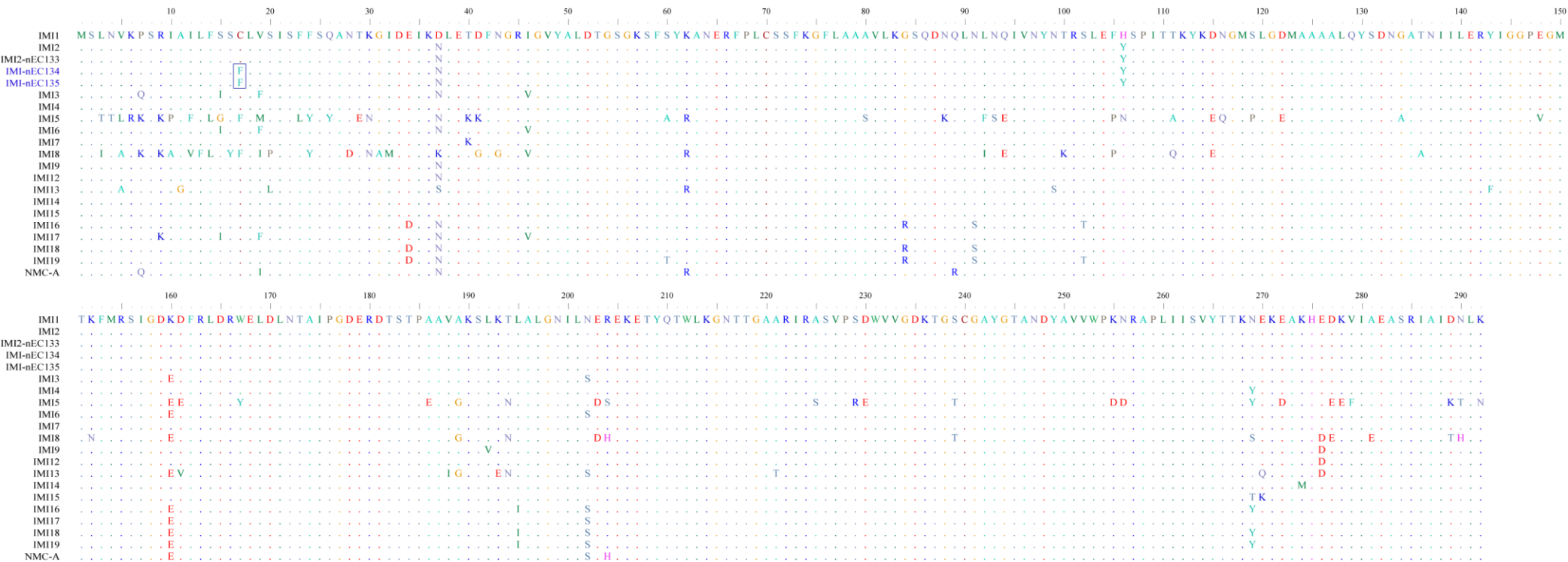

Supplement: Supplementary file 1 — Additional file 1. Figure 1: Alignment of IMI type carbapenem-hydrolyzing class A beta-lactamase amino acid sequences (see Figure 2 in the main text for accession numbers). Names of novel IMI alleles are marked in blue, cysteine to phenylalanine substitution is shown in the frame. [file 13756_2020_826_MOESM1_ESM.pdf]

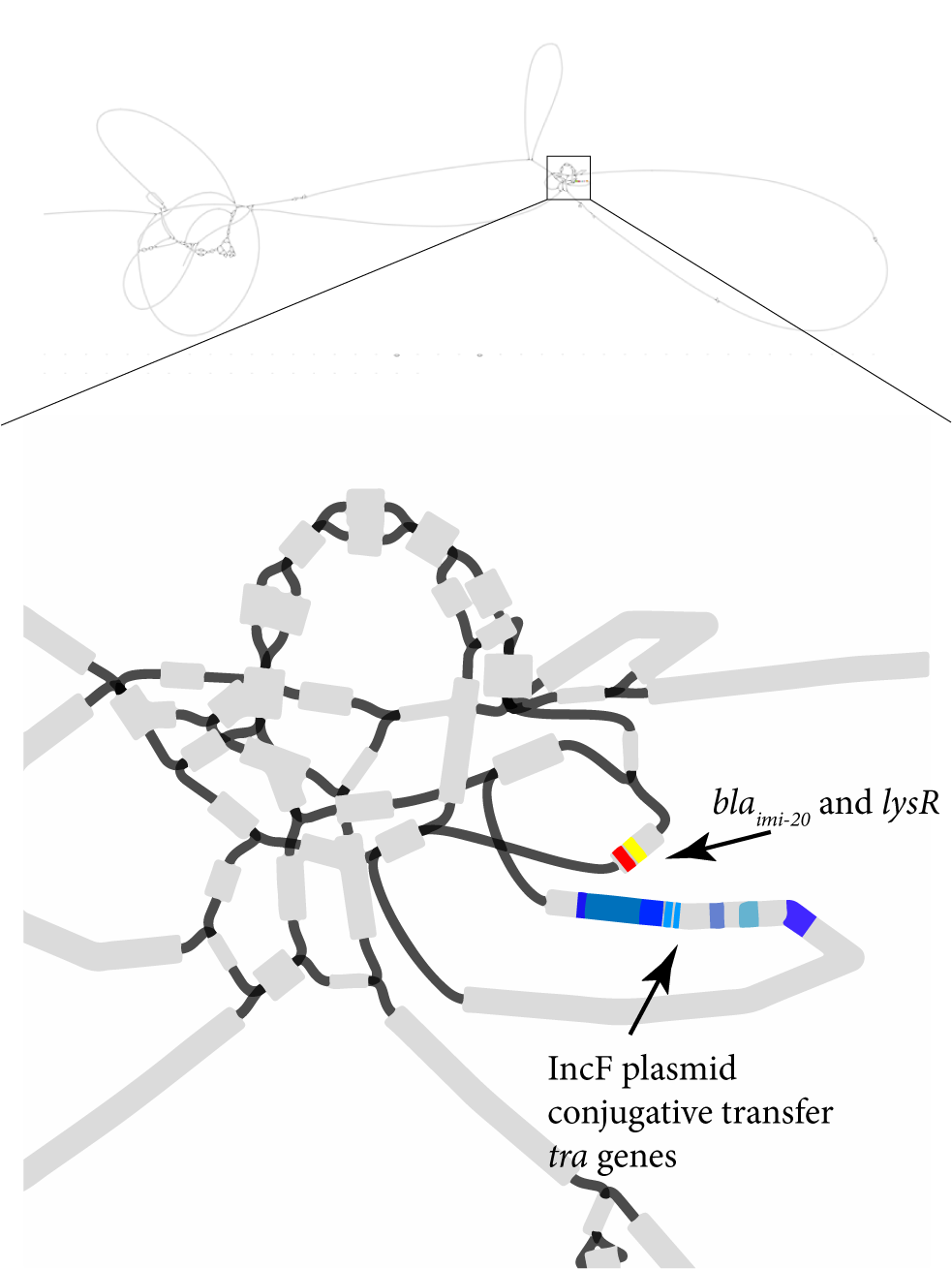

Supplement: Supplementary file 2 — Additional file 2. Figure 2: The link between blaMI-20 and traXIDTHBFNCUWICVBKEL genes of the IncF plasmid, based on the simplified de Bruijn assembly graph of the E. bugandensis nEC134 genome, generated by the SPAdes assembler and viewed in Bandage (the whole graph in the top panel and zoom in into the paths that contain the target genes in the bottom panel). The target genes are shown (blaIMI-20-red, lysR-yellow, traXIDTHBFNCUWICVBKEL-shades of blue). [file 13756_2020_826_MOESM2_ESM.tif]
